# Neutrophil elastase reduces secretion of secretory leukoproteinase inhibitor (SLPI) by lung epithelial cells: role of charge of the proteinase-inhibitor complex

**DOI:** 10.1186/1465-9921-9-60

**Published:** 2008-08-12

**Authors:** Anita L Sullivan, Timothy Dafforn, Pieter S Hiemstra, Robert A Stockley

**Affiliations:** 1 Department of Medicine, University of Birmingham, UK; 2 Department of Biosciences, University of Birmingham, UK; 3 Department of Pulmonology, Leiden University Medical Centre, The Netherlands; 4 Department of Respiratory Medicine, University Hospitals Birmingham, Birmingham, UK

## Abstract

**Background:**

Secretory leukoproteinase inhibitor (SLPI) is an important inhibitor of neutrophil elastase (NE), a proteinase implicated in the pathogenesis of lung diseases such as COPD. SLPI also has antimicrobial and anti-inflammatory properties, but the concentration of SLPI in lung secretions in COPD varies inversely with infection and the concentration of NE. A fall in SLPI concentration is also seen in culture supernatants of respiratory cells exposed to NE, for unknown reasons. We investigated the hypothesis that SLPI complexed with NE associates with cell membranes *in vitro*.

**Methods:**

Respiratory epithelial cells were cultured in the presence of SLPI, varying doses of proteinases over time, and in different experimental conditions. The likely predicted charge of the complex between SLPI and proteinases was assessed by theoretical molecular modelling.

**Results:**

We observed a rapid, linear decrease in SLPI concentration in culture supernatants with increasing concentration of NE and cathepsin G, but not with other serine proteinases. The effect of NE was inhibited fully by a synthetic NE inhibitor only when added at the same time as NE. Direct contact between NE and SLPI was required for a fall in SLPI concentration. Passive binding to cell culture plate materials was able to remove a substantial amount of SLPI both with and without NE. Theoretical molecular modelling of the structure of SLPI in complex with various proteinases showed a greater positive charge for the complex with NE and cathepsin G than for other proteinases, such as trypsin and mast cell tryptase, that also bind SLPI but without reducing its concentration.

**Conclusion:**

These data suggest that NE-mediated decrease in SLPI is a passive, charge-dependent phenomenon *in vitro*, which may correlate with changes observed *in vivo*.

## Background

SLPI is an efficient inhibitor of NE and other serine proteinases[1,2] and is found in high concentrations in secretions such as respiratory mucus[3]. *In vitro *it has antibacterial [4-6] and antifungal[7] properties and has been shown to prevent viral infection[8,9]. In addition it has anti-inflammatory properties distinct from inhibition of extracellular NE, that are potentially important in host defence and auto-immune conditions [10-14]. These properties suggest that SLPI may be important in diseases such as bronchiectasis and COPD that are characterized by neutrophilic inflammation and infection. SLPI should be protective in these conditions, and indeed patients with chronic bronchitis (CB) have a higher concentration of SLPI in lung secretions than healthy controls[15], probably because of submucosal gland hypertrophy and increased serous cell secretion. However the amount of SLPI found in the sputum of patients with CB and bronchiectasis decreased during exacerbations [16-18] and increased again in the stable state[16,18]. It was also lower in the stable state in sputum from patients with frequent exacerbations compared to those with infrequent exacerbations [19], suggesting that low concentrations of SLPI increase the risk of developing an exacerbation. In addition it was lowest in those patients with the greatest neutrophilic inflammation in the stable state[20], and lower in those who remained colonized in the stable state compared to those who cleared their infection[18,21]. This relation to infection has been confirmed in studies showing that SLPI was also reduced in vaginal secretions in the presence of bacterial infection [22].

In general, SLPI and NE concentration appear to be inversely related in sputum in COPD and CB[23] but the reason for this relationship *in vivo *is not understood. The genetics of patients with early-onset COPD have been investigated and no mutations, deletions or disease-associated polymorphisms in the SLPI gene have been described[24]. In our studies over 20 years, we have not identified any patients with an absence of SLPI in sputum. Therefore at present the concept that a primary deficiency of SLPI initiates inflammation or infection is not well supported by the available data.

A number of studies have described an inverse relationship between SLPI and NE *in vitro *using tissues ranging from primary nasal and bronchial epithelial cells, through a variety of cell lines, to isolated tracheal submucosal glands [25-28]. Since cell culture supernatant from cells exposed to NE has a lower concentration of SLPI protein than supernatant from cells not exposed to NE, neutrophilic inflammation may directly predispose to low SLPI *in vivo*. Previous *in vitro *work also included measurement of SLPI gene expression in cells exposed to NE and high levels were found to increase gene expression [26-29]. The fall in protein must therefore relate to alterations in post-transcriptional events. Immunohistochemical studies of primary nasal epithelial cells showed greater SLPI protein in cells treated with NE than those not treated, suggesting failure of secretion or redistribution of the protein rather than failure of synthesis[28]. Studies of NE-treated cells lysed using a detergent-based solution after removal of media demonstrated that the SLPI not found in cell culture media was in the cell lysate [27].

One mechanism that might explain the NE-induced redistribution of SLPI protein hinges on the positive charge of the NE molecule, which may enable it to associate with cell membranes [30]. Once associated with epithelial cell membranes, it could bind SLPI, although not as efficiently as in free solution[31], and would hence remove SLPI from cell supernatant. The known structures of NE[32] and SLPI[33] suggest that charged residues may remain exposed on the outside of each molecule when complexed with each other, so the NE-SLPI complex is likely to have a more potent positive charge and therefore is more likely than SLPI alone to associate with cell membranes and negatively-charged proteins.

Other possible mechanisms include failure to secrete, or binding of SLPI to other cell surface proteins exposed by NE. For example, SLPI has been shown to bind specifically to annexin II[34] and to scramblase[35], and these may potentially be upregulated or exposed in the presence of proteinases. It is known that there is a receptor specific for the complex of NE and α1 antitrypsin (α1 AT) [36], and therefore it is also possible that the SLPI-NE complex binds to a specific receptor. Finally, there may be a mechanism to import SLPI actively into cells. Studies with neutrophils[37], megakaryocytes and platelets[38] show SLPI inside cells, and a recent study indicated that macrophages can import exogenous SLPI into both the cytoplasm and nucleus[12].

In the present study, we have investigated the hypothesis that charge-related association of the SLPI-NE complex with cell membranes is the primary mechanism reducing SLPI concentration in cell culture supernatants from SLPI producing lung epithelial cells in the presence of NE. We have conducted experiments investigating the nature of the effect of NE on SLPI protein levels in cell culture supernatant, and pursued preliminary studies into the localization of SLPI in these cells when exposed to NE.

## Methods

### Proteinases and inhibitors

NE was purified from empyema fluid using the method of Martodam et al[39]. Absence of endotoxin contamination was confirmed with the Limulus amoebocyte assay (E-TOXATE, Sigma, UK). Activity of NE was measured using the synthetic chromogenic substrate N-succinyl-(ala)_3_-*p*-nitroanilide (Sigma, UK). Cathepsin G (CG) was similarly obtained from empyema fluid and activity was measured using N-succinyl-phe-pro-phe-*p*-nitroanilide (Bachem, UK). Porcine pancreatic elastase (PPE) was obtained from Sigma and the activity was measured using N-succinyl-(ala)_3_-*p*-nitroanilide. Trypsin was cell culture grade (Invitrogen) and activity was measured using N-benzoyl-L-arginine ethyl ester hydrochloride (BAEE) (Sigma). Human mast cell chymase and tryptase were obtained from Elastin Products Co. and activity was measured using N-succinyl-val-pro-phe-*p*-nitroanilide and Z-gly-pro-arg-*p*-nitroanilide respectively (both from Bachem). ZD0892, a small synthetic peptidyl trifluoromethylketone NE inhibitor with an affinity for NE similar to that of SLPI[40], was a gift from Zeneca Pharmaceuticals (Wilmington, USA) and inhibitory function was measured against a known amount of active NE using N-succinyl-(ala)_3_-*p*-nitroanilide. Recombinant human SLPI (rhSLPI) was a gift from Amgen and the concentration was confirmed using the SLPI ELISA (see below). Inhibitory activity was determined as for ZD0892.

### Measurement of SLPI and total protein

SLPI concentration was measured using the R&D Systems ELISA, a sandwich ELISA consisting of a mouse monoclonal antibody for capture and a horseradish peroxidase-conjugated polyclonal antibody for detection. Total protein was measured in microplates using the Bio-Rad assay (Bio-Rad, USA). The ELISA was validated using mixtures of SLPI and NE or CG in varying proportions to ensure that the proteinases did not interfere with quantification of SLPI. Briefly, recombinant human SLPI supplied as standard in the ELISA kit was mixed at varying molar ratios with NE and CG, incubated at 37°C for 30 minutes and the amount of SLPI recoverable was measured using the ELISA.

### Cell culture

A549 cells, a lung epithelial cell line derived from lung carcinoma, were obtained from the ECACC and cultured in 50/50 F10/DMEM (both from Gibco, UK) with 10% fetal calf serum (FCS) (Gibco). HepG2 cells, a liver epithelial cell line derived from liver carcinoma, were obtained from the ATCC. They were cultured in DMEM containing 10% FCS, antibiotic antimycotic solution, 150 mmol L-glutamine and MEM non-essential amino acids (all from Sigma). Primary human bronchial epithelial cells (PBEC) were obtained from Cambrex (UK) and cultured in the same supplier's recommended basal media with growth supplements according to their instructions. Experiments were carried out with cells between passage 3 and 8, using basal media only.

Most experiments were performed on A549 cells and selected experiments were repeated on PBEC or HepG2 cells in standard submerged monolayer culture. All experiments were performed in at least triplicate wells, and results are expressed as mean ± standard error of the mean (SEM) of at least 3 experiments. Cells were cultured to confluence in T75 or T25 flasks (Gibco) and plated out into 12 or 24 well plates (Gibco). Once confluent, the media were changed to serum free media (SFM) (50/50 F10/DMEM for A549 cells, DMEM alone for HepG2 cells and basal media without additives for PBEC). A549 cells and HepG2 cells were cultured in SFM for a further 24 h before experiments were performed. After treatment with the experimental conditions, supernatants were aspirated and stored at -70°C for later analysis for SLPI and protein concentrations. Cells were rinsed with PBS and harvested using trypsin to detach them and viability was assessed where appropriate using trypan blue exclusion. Statistical analysis was performed using SPSS version 12 for Windows.

### Experiments

#### 1. Dose response

A dose response experiment was undertaken to examine the relationship of NE concentration to the reduction of SLPI concentration in cell culture supernatants. The initial concentration of SLPI in the cell supernatants was not known at the outset of an experiment. Preliminary experimental data showed that the typical concentration of SLPI in supernatants of A549 cells after 24 h culture in SFM was between 0.5 and 2 nM (5–23.4 ng/ml). Cells were therefore incubated in SFM alone as control, or SFM containing NE from 0.5 to 5 nM. Initial experiments also included a control consisting of the buffer used to suspend the NE, in SFM at the same concentration as the highest concentration of NE used. Dose response experiments were repeated with CG and using PBEC with both proteinases.

#### 2. Effect of other proteinases

Proteinases such as PPE have, like NE, been shown to increase SLPI expression[29], although the effect on protein secretion has not been reported. PPE (which does not bind SLPI), human mast cell chymase, trypsin and human mast cell tryptase (which bind SLPI with varying affinity but do not carry a high positive charge) were used to assess whether the degree of binding to SLPI by the proteinase or its ability to associate with cell membranes by charge would modify the ability of the proteinase to reduce SLPI in the supernatant. The effect on SLPI secretion was compared for PPE, trypsin, chymase, tryptase and CG by incubating cells with varying concentrations of each proteinase for 24 h.

#### 3. Time course

The hypothesis of binding of the NE-SLPI complex to cell membranes because of positive charge suggested that addition of NE to conditioned media would cause removal of SLPI that had already been secreted. Confluent PBEC were cultured for 24 h in SFM and at the end of this period an aliquot was removed for baseline analysis and replaced with either SFM or NE at a concentration sufficient to achieve 10 nM in each well. After gentle mixing of the contents of the wells once with a 1 ml pipette, cells were incubated for a further 24 h and aliquots of 25 μl were taken at various time points within this period and stored at -70°C for analysis by ELISA. Similar experiments were repeated on A549 cells using both NE and CG, and with 1 nM rhSLPI in DMEM added to HepG2 cells followed by NE or SFM control. HepG2 cells were used as a control cell line that does not express or secrete SLPI, to demonstrate the effect of proteinases on SLPI concentration without any possibility of secreted SLPI contaminating the results.

#### 4. Effect of a synthetic NE inhibitor

Addition of NE inhibitors to culture media containing NE has been shown to abrogate the fall in SLPI protein[27]. If binding of NE to SLPI is important in the mechanism of the fall in SLPI protein, then the timing of the addition of the inhibitor will be equally important. NE was incubated for 30 min at 37°C with a tenfold excess of ZD0892. The mixture of NE and ZD0892 was added to the supernatant of A549 cells that had been serum-starved for 24 h, to achieve a final concentration of 10 nM NE and 100 nM ZD0892. This was compared with NE 10 nM added 10 minutes prior to the addition of 100 nM ZD0892, and ZD0892 100 nM added 10 minutes prior to the addition of NE 10 nM. Control cells were treated with NE 10 nM alone, ZD0892 100 nM alone, or SFM alone. All mixtures were incubated for a further 10 minutes and the supernatants were then aspirated and stored at -70°C for analysis by ELISA.

#### 5. Localization of SLPI in cell culture system

Given that many proteins associate non-specifically with charged surfaces such as cell culture plastic, we speculated that the NE-SLPI complex may associate more with cell culture materials than SLPI alone. This was tested by comparing the concentration of SLPI in solutions added to empty tissue culture plates with the concentration of SLPI in solutions added to wells containing confluent cells, with and without NE. SLPI was added to SFM to achieve a concentration of 1 nM, and the solution was incubated in 12 well plates for 10 minutes, a baseline aliquot was taken for measurement of SLPI and replaced with SFM or NE to achieve a final enzyme concentration of 10 nM. After a further period of incubation the contents of the wells were harvested for measurement of SLPI concentration. In order to block the effect of non-specific binding, both 12 well plates containing cells and 12 well plates without cells were also incubated with media containing 1% w/v human serum albumin (HSA) and SLPI at 1 nM. The effect of 10 nM NE was assessed as before in both sets of plates. Plates were also coated with HSA prior to addition of cells, and the above experiments were repeated using plates with or without A549 cells. Finally in cell-free conditions, the effect of 1% Tween 20 (polyoxyethylene sorbitan monolaurate, Sigma) was assessed in the same way.

#### 6. Two compartment model

SLPI is secreted both basally and apically by cells *in vitro*[41], and *in vivo *epithelial and glandular cells might be exposed to NE release from neutrophils at both sites during neutrophil migration. To examine whether this may influence the secretion of SLPI, a two compartment model was studied. Transwells (Transwell PET 0.4 μm pore size 12 mm inserts, Corning Life Sciences) were coated with human placental collagen (Sigma) at 50 μg/ml and dried in air. A549 cells were added to the upper compartment in 500 μl media, with 1.5 ml media in the lower compartment. As soon as the cells appeared as a confluent monolayer, media were replaced by SFM at the same volumes. After a further 24 h incubation, reagents were added to upper and lower compartments. Triplicate wells received either SFM alone in both compartments or NE 10 nM in one compartment with SFM in the other. Supernatants were aspirated after a further 10 minutes incubation, and stored at -70°C for later analysis for total protein and SLPI concentration.

#### 7. Theoretical modelling of the external charge of the complex between SLPI and proteinases

The modelling procedure involved the use of the X-ray crystal structure of SLPI with chymotrypsin (structure file kindly provided by Professor Wolfram Bode as described previously[33]) as a template to construct analogous complexes with other proteinases. X-ray crystal structures of NE (PDB ID 1H1B; [42]), CG(PDB ID 1T32;[43]), trypsin (PDB ID 1TX6;[44]), mast cell chymase (PDB ID 1T31;[43]) and mast cell tryptase (PDB ID 2BM2;[45]) were obtained from the Research Collaboratory for Structural Bioinformatics Protein Data Bank[46]. These structures were then superimposed on the proteinase in the SLPI-proteinase complex using SWISSPDB[47]. The values for the original proteinase (chymotrypsin) were then deleted and the new complex saved. SLPI alone was obtained by deleting chymotrypsin from the original structure file. Electrostatic surfaces were generated using SWISSPDB with values of -3.00, 2.00 and 8.00 for the red, white and blue extremes of the spectrum.

## Results

**Figure 1:**
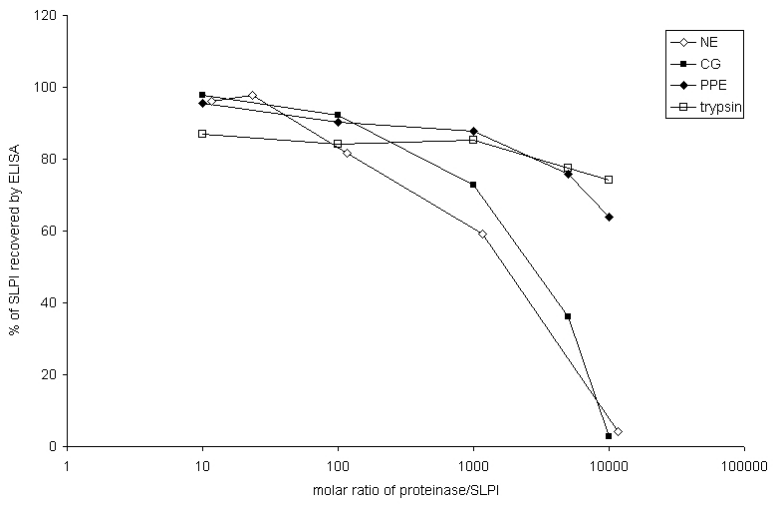
**Validation of the ELISA for measuring SLPI with proteinases**. Recombinant human SLPI was mixed with varying concentrations of NE and assayed by ELISA to assess the recovery of SLPI in complex with the proteinase and in the presence of excess proteinase. The molar ratio of proteinase to SLPI is given along the x axis, and the percentage of SLPI recovered is shown on the y axis. The figure shows the results obtained with NE, CG, PPE and trypsin. The ELISA was affected by a large molar excess of proteinase, but not at the molar ratios relevant to this paper (< 10:1).

The ELISA confirmed SLPI concentration was fully recoverable at the range of concentrations of NE and CG used in these experiments. However at very high molar ratios of proteinase to inhibitor (> 100:1), there was a fall in quantification which was attributed to the effects of unopposed proteinase activity on the antibody used in the ELISA (Figure 1).

### 1. Dose-dependent effect of NE on SLPI in A549 supernatants

**Figure 2:**
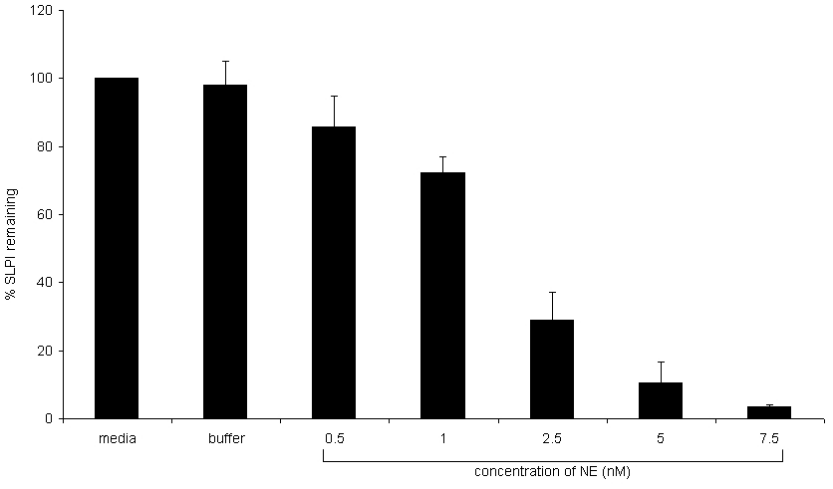
**Effect of neutrophil elastase (NE) on SLPI in supernatants from cultured A549 cells**. A549 cells were cultured for 24 h with NE at the concentrations shown, with an equivalent concentration of the buffer alone, or in a serum free media control. The y axis shows the average of the SLPI secreted in each condition as a percentage of the SLPI secreted by control cells (% SLPI release). Error bars represent standard error of the mean (SEM). N = 3–5 separate experiments for all except buffer control (N = 2). A dose-related reduction in SLPI supernatant concentration was seen with NE (statistically significant from 2.5 nM) (p = 0.006, one way ANOVA).

NE at the concentrations used in these experiments did not affect cell viability (by trypan blue exclusion, data not shown). The concentration of SLPI after 24 h showed a dose related fall with NE treatment from 1.36 ± 0.37 nM at 0.5 nM to 0.20 ± 0.14 nM at 5 nM, whilst media control contained 1.63 ± 0.31 nM (p = 0.006, one way ANOVA) (Figure 2). Similar results were obtained when the experiments were repeated with A549 cells using CG and with PBEC using NE (data not shown).

**Figure 3:**
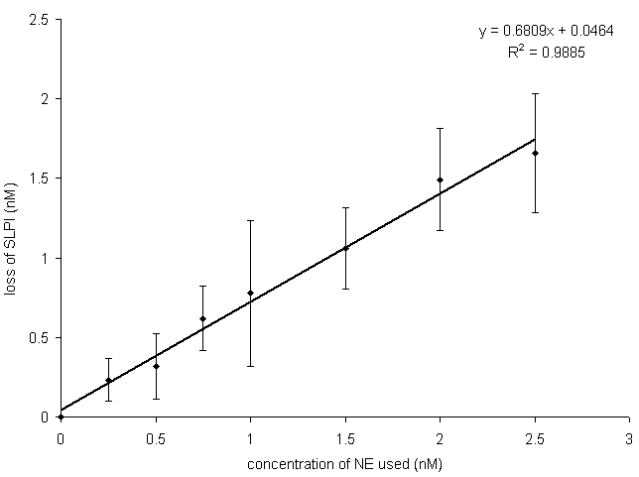
**Relationship between NE and SLPI decrease in the supernatant of A549 cells**. The total NE-induced decrease in SLPI concentration, calculated from the experiment shown in Figure 2, was plotted against the concentration of NE used and a linear relationship found (Pearson correlation coefficient 0.846, p < 0.001).

Using a dose range from 0–2.5 nM NE, a linear relationship was seen between the difference in SLPI concentration between treated and control cells, and the amount of NE (Figure 3). This relationship suggests about 1.4 molecules of NE are required to remove 1 molecule of SLPI from cell culture media.

### 2. Effect of other proteinases

**Figure 4:**
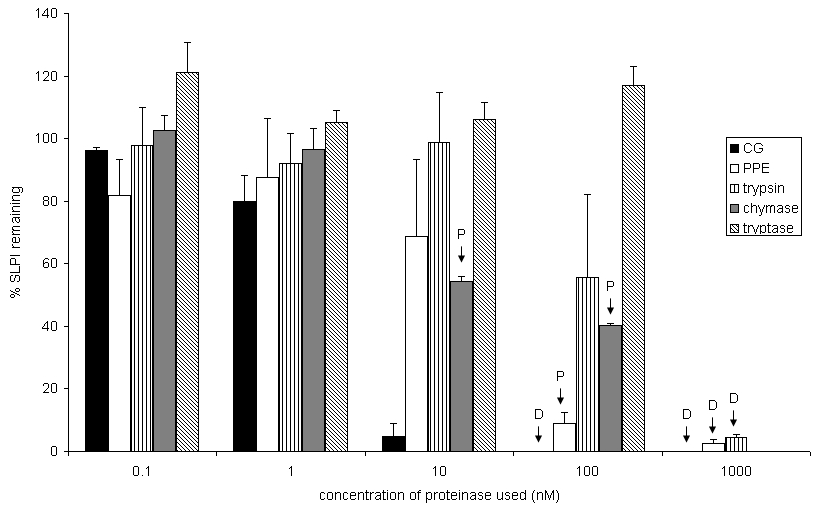
**Effect of other proteinases on SLPI concentration in A549 cells**. A549 cells were cultured with varying concentrations of each proteinase for 24 h. The concentration of SLPI, expressed as a percentage of the concentration in serum free media, is shown on the *y *axis with the SEM represented by error bars. P indicates that the cells exhibited morphological changes of partial detachment, 'rounding up' and losing attachment to each other but remaining adherent to the culture plate, whilst D indicates that the cells detached completely from the culture plate. The effect on SLPI concentration was only independent of cell morphology for CG. Values given are mean ± SEM for at least 3 experiments.

At high concentrations of proteinases, all cells detached from the wells although cell viability was not affected during the duration of the experiment as assessed by trypan blue exclusion (data not shown). At these higher proteinase concentrations, SLPI concentration fell sharply. CG caused a dose-dependent fall in SLPI concentration (79.9 ± 8.2% of control values (p < 0.05) at 1 nM, 4.7 ± 4.3%, 0.02 ± 0.0% and 0.07 ± 0.1%, (p < 0.001) at 10 nM, 100 nM and 1000 nM respectively), but had no effect at 0.1 nM (96.2 ± 0.8% control) while SLPI concentration fell significantly only at high concentrations for PPE (9.0 ± 3.5% at 100 nM, 2.6 ± 1.3% at 1000 nM, p < 0.005) and trypsin (4.5 ± 0.8% at 1000 nM, p < 0.005), at which cell morphology was affected. Mast cell chymase caused a significant reduction in SLPI secretion at concentrations from 10–100 nM. This was associated with a marked change in cell morphology: loss of cell-cell contact but no detachment. At lower concentrations, with normal cell morphology, there was no effect on SLPI secretion. Mast cell tryptase did not affect either SLPI concentration or cell morphology at any dose (Figure 4).

### 3. Time course of the effect of NE

**Figure 5:**
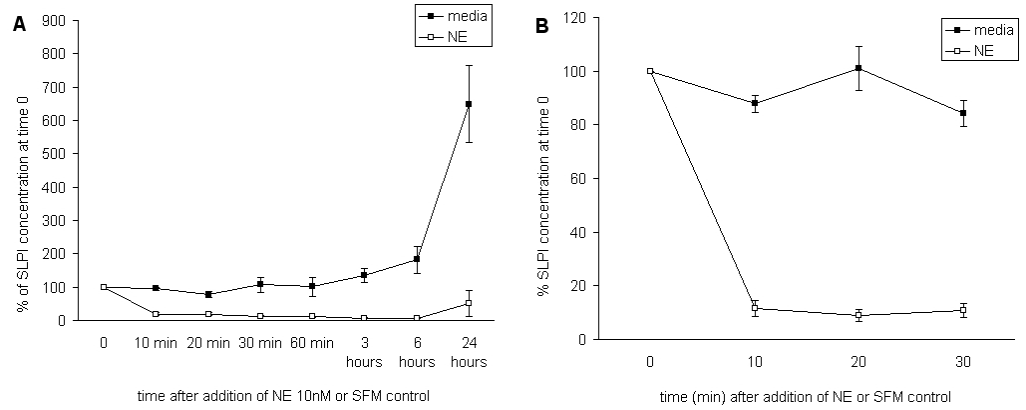
**Time course of the effect of NE on SLPI concentration**. Figure 5a: PBEC were cultured in basal media for 24 h, and then an aliquot of media was removed as baseline and replaced with the same volume of media control or media containing NE at a concentration sufficient to achieve a concentration of 10 nM in the wells. Further aliquots were taken at the times shown, and SLPI concentration was measured by ELISA. The figure shows the results from 3 experiments. The y axis gives the concentration of SLPI as % of the baseline value for triplicate wells treated with basal media or NE. (Error bars indicate SEM.) The concentration of SLPI fell at the earliest time point studied in wells treated with NE but remained stable in the media controls. Over 24 h the concentration in media controls rose in accordance with the steady state concentrations predicted, but remained low in NE-treated wells. At 24 h, some SLPI was present in the media of NE-treated cells but this was substantially lower than the media controls (p < 0.001 Wilcoxon signed ranks test). b: HepG2 cells were cultured in 12 well plates and, after rinsing with PBS, media containing 1 nM SLPI was added to the cells. After a few minutes equilibration, a baseline aliquot was removed and replaced with either media control or NE to achieve a final concentration of 10 nM. Further aliquots were taken at the time points shown. In the media controls it remained stable throughout, but the concentration in NE-treated wells fell significantly at 10 minutes and remained low thereafter (p < 0.001 Wilcoxon signed ranks test).

Several groups have demonstrated that the presence of NE reduces the amount of SLPI in cell supernatants after periods of culture ranging from 1 h to 48 h[26,27]. When NE was added to wells containing A549 cells following 24 hours incubation (therefore containing substantial amounts of SLPI), a rapid fall in SLPI concentration was seen at the earliest time point tested (10 min). The results were similar with PBEC (Figure 5a), A549 cells (data not shown) and HepG2 cells (Fig 5b). CG also caused a decrease in supernatant SLPI from media of A549 cells within 10 min (control wells 102.36 ± 3.75% of baseline, wells treated with CG 10 nM 1.50 ± 0.27% baseline at 10 minutes). Subsequent experiments using A549 cells showed that NE already caused a detectable decrease in SLPI within 2 min (data not shown).

### 4. Effect of synthetic NE inhibitor

**Figure 6:**
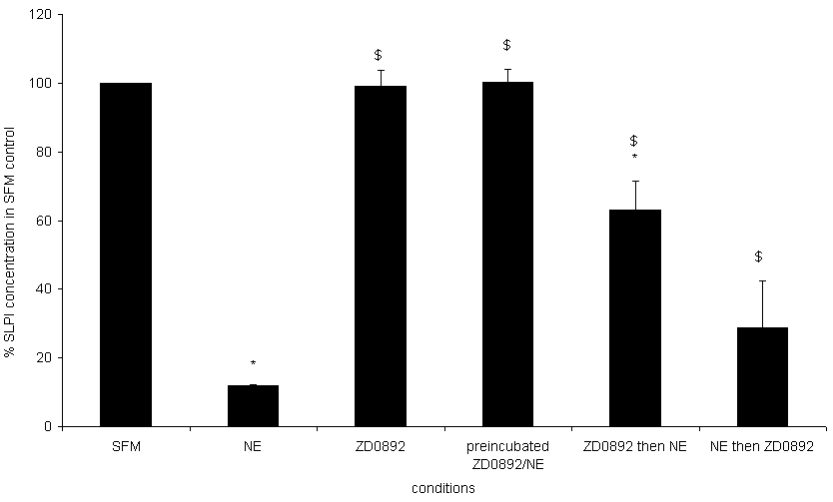
**Timing of the addition of inhibitor to NE affects concentration of SLPI**. The diagram shows the concentration of SLPI in the media of wells treated according to the conditions shown on the *x *axis. The concentration of SLPI is expressed as % of the concentration in control wells. * indicates significant difference from control wells, $ indicates significant difference from NE-treated wells. Values shown are mean ± SEM for 3 experiments. Prior incubation of NE with a tenfold excess of ZD0892 completely abrogated the effect of NE on SLPI concentration. When NE and ZD0892 were added separately, there was a fall in SLPI concentration which was less pronounced than that seen with NE alone, but still significantly different from both media control and NE alone. Although the average fall was greater when NE was added before rather than after ZD0892, this difference was not statistically significant.

ZD0892 alone did not affect SLPI concentration in A549 supernatants (99.12 ± 4.7% of media control). The concentration of SLPI in NE-treated wells fell to 12.05 ± 2% of control values (p < 0.001 compared to media control). When NE and ZD0892 were pre-incubated, the effect of NE on SLPI was abrogated (100.2 ± 3.7% media control, p < 0.005 compared to NE alone). Addition of NE after ZD0892 caused some decrease in SLPI concentration (to 63.0 ± 8.5% of media control, p < 0.05 compared to media control) but the fall in SLPI concentration was less than with NE alone (p < 0.05). Addition of NE before ZD0892 caused a fall in SLPI concentration (to 28.7 ± 13.7% control, p < 0.05 compared to media control), which was not significantly different from NE alone. The difference between the results for NE and ZD0892 added in either order was not statistically significant. These experiments are summarised in Figure 6.

### 5. Attachment of SLPI to plastic surfaces

We next explored whether the loss of SLPI in the presence of NE required the presence of cells, or whether it would occur in cell-free conditions. We added SLPI to empty tissue culture plate wells with or without NE. There was a marked loss of SLPI from the empty tissue culture plates as only about 1% of the expected amount was recovered from control wells and therefore an additional effect of NE could not be identified. Further investigation demonstrated that there was a large loss of SLPI when diluted in SFM in standard 20 ml polystyrene tubes, and a further loss in tissue culture plates, which was attributed to charge-related non-specific binding to plastic (data not shown). This may have explained the observed decrease in SLPI in cells treated with high concentrations of trypsin or PPE that caused detachment, since this would have exposed culture plastic which would be capable of binding the previously secreted SLPI, resulting in a fall in SLPI concentration in the supernatant.

**Figure 7:**
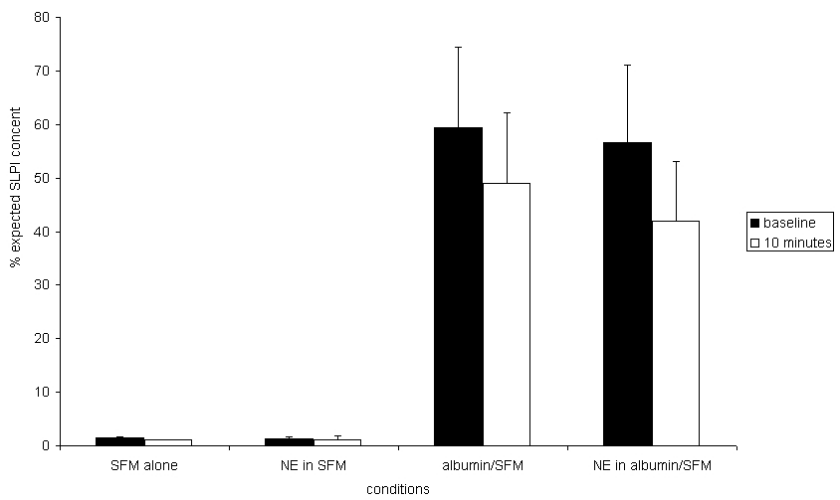
**Protection by albumin against non-specific binding of SLPI to plastic**. The diagram shows the concentration of SLPI in SFM added to tissue culture plates without cells, before addition of NE or control (baseline) and 10 min afterwards. Parallel wells used SFM containing human serum albumin at 1 mg/ml. Data shown are the mean results from 3 experiments with SEM represented by the error bars. Without albumin, almost all the SLPI was lost regardless of whether NE was added. Albumin provided partial protection against loss of SLPI at baseline and after addition of NE.

Use of polypropylene plates and siliconised glass culture systems did not offer more than partial protection against this loss of SLPI (data not shown) and cells did not grow satisfactorily without a substrate on siliconised glass. Addition of HSA 1% w/v to culture media, or coating of tissue culture plates with HSA or human placental collagen prior to plating out cells, partially reduced the non-specific loss of SLPI from culture media (data not shown). Furthermore the presence of HSA in solution partially inhibited the effect of NE in reducing SLPI concentration in cell-free systems (Figure 7), and similarly in plates coated with HSA prior to plating out cells (data not shown). Addition of 1% Tween to cell-free systems completely prevented loss of SLPI when incubated without NE, but did not prevent almost total loss of SLPI in the presence of NE (data not shown).

### 6. Two compartment model

**Figure 8:**
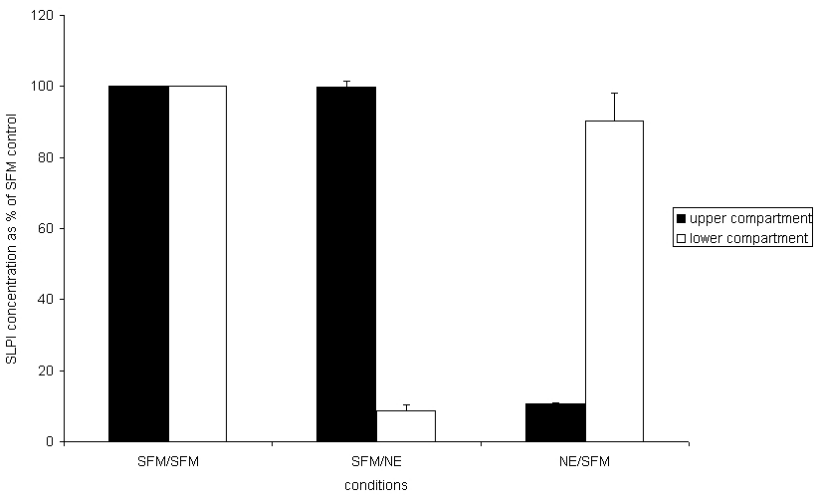
**The effect of NE on SLPI concentration in media of cells grown on Transwells**. The diagram shows the concentration of SLPI in each compartment of A549 cells grown in Transwells, relative to the concentration in control wells. Wells were treated with either 10 nM NE or serum free media (SFM) alone for 10 minutes, and the concentration of SLPI was then determined by ELISA. Upper and lower compartment results for the same well are adjacent; the solid bars indicate upper compartments and the open bars indicate lower compartments. SFM/NE indicates that NE was applied to the lower compartment only whilst NE/SFM indicates that NE was applied to the upper compartment only. The concentration of SLPI in the NE-treated compartments fell significantly (p = 0.001 by independent t-test) from 100% to 10.6 ± 0.3% (NE added to upper compartment) and 8.6 ± 1.9% (NE added to lower compartment) whilst there was no difference in concentration of the paired SFM-treated compartments (99.9 ± 1.5% and 90.31 ± 7.8% of control wells respectively).

Supernatant from the upper compartments of Transwells on which A549 cells were cultured contained about fourteen times as much SLPI as the lower compartments. The difference may be due to polarized secretion of SLPI, or might be accounted for by SLPI binding to the plastic of the cell culture plates in the lower compartment, which had not been treated to prevent non-specific binding and contained no cells. Addition of NE to the upper compartment caused reduction in SLPI concentration to 10.6 ± 0.3% of control values, and in the lower compartment it caused a reduction to 8.6 ± 1.9% of control. SLPI concentrations in the control-treated compartment were not affected (Figure 8).

### 7. Theoretical modelling of SLPI in complex with proteinases

**Figure 9:**
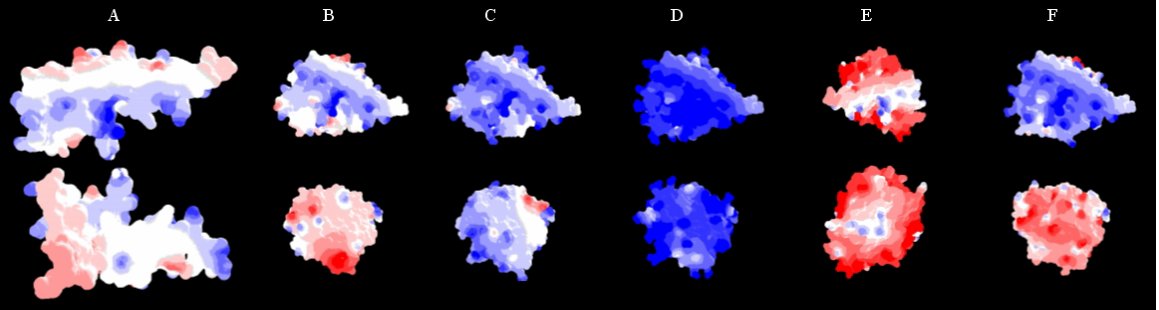
**Predicted charge of the complex of SLPI with various proteinases**. Modelling of the SLPI molecule alone (A) and in complex with various proteinases (B-F): blue indicates positive charge, white is neutral and red indicates negative charge. All models are shown in the same orientation with views of opposite sides. The SLPI molecule was predominantly neutral with some positively-charged areas. Cathepsin G (D) formed the most positively-charged complex, followed by NE (C). Chymase (F) was strongly positively-charged on one surface only. Trypsin (B) had weak positive and negative charge on different surfaces, and tryptase (E) was strongly negatively-charged.

Figure 9 shows the SLPI molecule alone (Fig 9a) and SLPI-proteinase complexes (Fig 9b–f). Using colour to indicate charge (red for negative, blue for positive), it can be seen that both NE and CG produced a highly positively-charged complex with SLPI. Mast cell chymase and trypsin varied in charge on the two aspects shown whereas mast cell tryptase was highly negatively-charged on both aspects. PPE was not modelled for this experiment because it does not bind SLPI. The results suggest that SLPI in complex with CG and NE would be predicted to associate more with negatively-charged surfaces than either free SLPI, or SLPI complexed with other proteinases, because of the greater positive charge.

## Discussion

The results from the present study show that both NE and CG decrease SLPI released into culture supernatants from lung epithelial cells. The dose response of the proteinases was linear, and the effect was almost immediate. Experiments using a synthetic NE inhibitor showed that when the inhibitor was added together with NE, the NE-mediated decrease in SLPI was completely prevented. In contrast, when the inhibitor was added after NE had been applied to the cells it only partially prevented a reduction in SLPI. Experiments using a Transwell model showed that the effect of NE on SLPI required direct contact between these molecules, suggesting that direct binding of NE or CG to SLPI was involved.

Only the strongly cationic proteinases NE and CG were able to reduce SLPI concentration at almost molar equivalent doses. The other proteinases only caused reduction in SLPI when used at concentrations sufficient to alter cell morphology and/or cause detachment, which probably leads to non-specific binding to the tissue culture plastic. Theoretical modelling of surface charge supported the concept that an NE-SLPI or CG-SLPI complex would have a greater positive charge than SLPI alone or in complex with the other proteinases studied. Therefore it seems likely that the reduction in SLPI concentration in the presence of proteinases was mediated by charge-related binding to other structures.

Previous studies on the effect of NE on SLPI *in vitro *have shown a decrease in SLPI concentration with a variety of cells and tissues [25-27], but only two studies have attempted to explore this further. The study by Marchand *et al*[28] used immunohistochemistry to examine nasal explanted epithelial tissue exposed to NE and found greater signal than in the control tissues not exposed to NE. The study by van Wetering *et al*[27] used PBEC and again found a dose-dependent effect, present from a few hours after exposure of cells to NE, which could be inhibited by co-administration of an inhibitor (α1 AT). Analysis of cell lysates indicated that SLPI became cell-associated in the presence of NE rather than in the culture media, and hence suggested a block in secretion or redistribution of the protein rather than an effect on synthesis. These studies did not pursue more detailed analysis of the relationship between NE and SLPI concentration and the possible mechanism responsible for this relationship. In addition, none of the studies in the literature has controlled for non-specific binding of SLPI to tissue culture materials. Studies on the ability of SLPI to protect fibronectin from degradation by NE showed that SLPI could associate with this substrate through ionic interactions [48] and examination of human lung tissue showed that SLPI was associated with elastin fibres[49], again suggesting non-specific binding. SLPI binds non-specifically to many large molecules such as mucins[50] and DNA[51], however, to our knowledge no studies have examined whether the NE-SLPI complex is more likely to associate with cells or substrates than SLPI alone.

Our initial validation studies demonstrated that in the presence of a molar excess of NE or CG, detection of SLPI by ELISA was not affected. The studies described here did not examine the possibility of cleavage or degradation of SLPI by excess of proteinase, but even if this happened, the cleavage products would appear to be detected fully by the ELISA, suggesting that the monoclonal capture antibody recognises an epitope not affected by proteolysis, and that the polyclonal detection antibody also recognises preserved sites. Incubation with samples containing excessive amounts of NE may however expose the capture antibody to proteolytic degradation, and this may be the explanation for the fall in detection at high concentrations of NE, rather than degradation of SLPI by NE. Since the validation studies used recombinant rather than endogenous SLPI and were conducted in assay buffer rather than conditioned cell culture media, it cannot be guaranteed that the ELISA would perform as reliably in experimental conditions. However in the published studies previously mentioned, the same effect of dose-dependent reduction of SLPI in the cell culture environment was shown by systems using a variety of different antibodies for detection of SLPI, suggesting that the effect is real rather than a systematic measurement error. Possible effects on the ELISA from complex formation or cleavage of SLPI therefore cannot fully explain the complete loss of signal of SLPI in the presence of cells treated with NE. Nevertheless, our experiments do not conclusively demonstrate that SLPI bound to cells in presence of NE is biologically active. Furthermore, it needs to be noted that the difficulties in avoiding non-specific binding of SLPI to the plastic components of the cell culture system are not fully countered in these studies. If the hypothesis that binding of SLPI to NE causes adherence to cells by charge interactions is valid, then the same mechanism could apply to any negatively-charged surface, such as plastic cell culture components which were also shown to bind free SLPI avidly. NE did appear to increase non-specific binding of SLPI to plastic, because when detergent was used as a blocking agent, SLPI concentration fell only in the presence of NE (suggesting that the higher ionic charge of the NE-SLPI complex was able to overcome the concentration of detergent used). The ELISA system contains strong buffering agents that must be presumed to protect against loss of free SLPI. The validation studies suggest that they are sufficient to protect also against loss of the NE-SLPI complex. However it cannot be presumed that there was not loss of SLPI to plastic during the cell culture process, harvesting of supernatant and storing of the samples prior to preparation of the ELISA, and that this loss of SLPI was not enhanced in the presence of NE solely by increased binding to plastic rather than to cell membranes. Proof of the concept of binding of SLPI to cells would require a different experimental approach, and therefore the experiments presented here cannot claim to prove that the effect is biological and relevant to the *in vivo *situation. The location of the lost SLPI protein remains uncertain, although preliminary immunofluorescence studies in our laboratory suggested at least in part that the SLPI does become internalised (data not shown). Further studies, using SLPI bound to a fluorescent or radioactive label that does not impede binding to NE, will be required to confirm this.

There is a likely biological relevance of the phenomenon of free SLPI associating with cell membranes and extracellular matrix proteins because this would allow the lung to establish a protective antiproteinase coat over structures that would be at most risk of damage by free NE. The major function of NE is probably intracellular killing of bacteria within the phagolysosome. Where neutrophils are migrating through the lung, some local NE activity (from a released azurophil granule or released from cell membrane association) may possibly be beneficial to this process. Free NE in lung secretions however is believed to be uniformly deleterious. Where this occurs, it would be neutralized to some extent by the presence of SLPI bound to vulnerable structures. There may be some value in the SLPI-NE complex associating with these structures. If the NE-SLPI complex prevented further association of NE with extracellular matrix substrates more effectively than SLPI alone (because of its higher positive charge), it would leave the free NE in epithelial lining fluid to be inactivated by α1 AT, which the latter cannot do once NE is bound to substrate[52]. There are no published data on the avidity of binding of the NE-SLPI complex to cell membranes or matrix substrates. However it is known that there is a strong association between NE and neutrophil cell membranes[30], and between SLPI and fibronectin[48], based on the ionic strength or pH required to dissociate them. The ionic charge of the NE-SLPI complex has never been measured, because the binding of these two strongly cationic molecules is sufficiently reversible that standard electrophoretic methods cause them to dissociate. It is also unknown how stable the complex would be when membrane-bound. It is possible that membrane-bound complex might be able to dissociate, or be passively internalised during membrane cycling, or actively internalised by endocytosis or phagocytosis, probably requiring a specific receptor. Internalisation of the complex might generate biological responses central to lung inflammation. Further studies will clearly be required to explore these possibilities.

## Conclusion

In summary, the experiments described in this paper support the hypothesis that the association of SLPI in complex with NE to negatively-charged structures leads to its removal from cell culture media, and that this effect is a passive biochemical process, probably dependent on charge. It is likely that this effect occurs with cell membranes as well as non-biological components, but this has yet to be confirmed. Future experiments should include measures to counter the ability of SLPI to bind non-specifically to a variety of structures, particularly in the presence of NE or CG.

## Abbreviations

SLPI: Secretory leukoproteinase inhibitor; NE: Neutrophil elastase; COPD: Chronic obstructive pulmonary disease; CB: Chronic bronchitis, α1 AT: alpha 1 antitrypsin inhibitor; CG: Cathepsin G, PPE: Porcine pancreatic elastase; ELISA: enzyme linked immunosorbent assay; ECACC: European Collection of Cell Cultures; DMEM: Dulbecco's modified Eagle's medium; FCS: Fetal calf serum, ATCC: American Type Culture Collection; MEM: Minimum essential medium; PBEC: primary bronchial epithelial cells; PBS: Phosphate-buffered saline; SEM: Standard error of the mean; SFM: Serum free media, HSA: Human serum albumin, DNA: deoxyribonucleic acid.

## Competing interests

The authors declare that they have no competing interests.

## Authors' contributions

AS carried out the experimental work and wrote the manuscript. TD carried out the molecular modelling studies and produced the illustrations. PH conceived the hypothesis, advised on experimental work and assisted in drafting the manuscript. RS supervised the experimental work and assisted in drafting the manuscript. All authors read and approved the final manuscript.

## Availability & requirements





## References

[B1] Thompson RC, Ohlsson K (1986). Isolation, properties, and complete amino acid sequence of human secretory leukocyte protease inhibitor, a potent inhibitor of leukocyte elastase. Proc Natl Acad Sci U S A.

[B2] Smith CE, Johnson DA (1985). Human bronchial leucocyte proteinase inhibitor. Rapid isolation and kinetic analysis with human leucocyte proteinases. Biochem J.

[B3] Ohlsson K, Tegner H, Akesson U (1977). Isolation and partial characterization of a low molecular weight acid stable protease inhibitor from human bronchial secretion. Hoppe Seylers Z Physiol Chem.

[B4] Hiemstra PS, Maassen RJ, Stolk J, Heinzel-Wieland R, Steffens GJ, Dijkman JH (1996). Antibacterial activity of antileukoprotease. Infect Immun.

[B5] Singh PK, Tack BF, McCray PB, Welsh MJ (2000). Synergistic and additive killing by antimicrobial factors found in human airway surface liquid. Am J Physiol Lung Cell Mol Physiol.

[B6] Fahey JV, Wira CR (2002). Effect of menstrual status on antibacterial activity and secretory leukocyte protease inhibitor production by human uterine epithelial cells in culture. J Infect Dis.

[B7] Tomee JF, Hiemstra PS, Heinzel-Wieland R, Kauffman HF (1997). Antileukoprotease: an endogenous protein in the innate mucosal defense against fungi. J Infect Dis.

[B8] Hocini H, Becquart P, Bouhlal H, Adle-Biassette H, Kazatchkine MD, Belec L (2000). Secretory leukocyte protease inhibitor inhibits infection of monocytes and lymphocytes with human immunodeficiency virus type 1 but does not interfere with transcytosis of cell-associated virus across tight epithelial barriers. Clin Diagn Lab Immunol.

[B9] Beppu Y, Imamura Y, Tashiro M, Towatari T, Ariga H, Kido H (1997). Human mucus protease inhibitor in airway fluids is a potential defensive compound against infection with influenza A and Sendai viruses. J Biochem.

[B10] Taggart CC, Greene CM, McElvaney MG, O'Neill S (2002). Secretory leucoprotease inhibitor prevents LPS-induced IkB alpha degradation without affecting phosphorylation or ubiquitination. J Biol Chem.

[B11] Ding A, Thieblemont N, Zhu J, Jin F, Zhang J, Wright S (1999). Secretory leukocyte protease inhibitor interferes with uptake of lipopolysaccharide by macrophages. Infect Immun.

[B12] Taggart CC, Cryan SA, Weldon S, Gibbons A, Greene CM, Kelly E, Low TB, O'Neill SJ, McElvaney NG (2005). Secretory leucoprotease inhibitor binds to NF-kB binding sites in monocytes and inhibits p65 binding. J Exp Med.

[B13] Greene CM, McElvaney NG, O'Neill SJ, Taggart CC (2004). Secretory leucoprotease inhibitor impairs toll-like receptor 2- and 4-mediated responses in monocytic cells. Infect Immun.

[B14] Song X, Zeng L, Jin W, Thompson J, Mizel DE, Lei K, Billinghurst RC, Poole AR, Wahl SM (1999). Secretory leukocyte protease inhibitor suppresses the inflammation and joint damage of bacterial cell wall-induced arthritis. J Exp Med.

[B15] Stockley RA, Morrison HM (1990). Elastase inhibitors of the respiratory tract. Eur Respir J.

[B16] Hill AT, Campbell EJ, Bayley DL, Hill SL, Stockley RA (1999). Evidence for excessive bronchial inflammation during an acute exacerbation of chronic obstructive pulmonary disease in patients with alpha(1)-antitrypsin deficiency (PiZ). Am J Respir Crit Care Med.

[B17] Dijkman JH, Kramps JA, Franken C (1986). Antileukoprotease in sputum during bronchial infections. Chest.

[B18] White AJ, Gompertz S, Bayley DL, Hill SL, O'Brien C, Unsal I, Stockley RA (2003). Resolution of bronchial inflammation is related to bacterial eradication following treatment of exacerbations of chronic bronchitis. Thorax.

[B19] Gompertz S, Bayley DL, Hill SL, Stockley RA (2001). Relationship between airway inflammation and the frequency of exacerbations in patients with smoking related COPD. Thorax.

[B20] Hill AT, Bayley D, Stockley RA (1999). The interrelationship of sputum inflammatory markers in patients with chronic bronchitis. Am J Respir Crit Care Med.

[B21] Hill AT, Campbell EJ, Hill SL, Bayley DL, Stockley RA (2000). Association between airway bacterial load and markers of airway inflammation in patients with stable chronic bronchitis. Am J Med.

[B22] Draper DL, Landers DV, Krohn MA, Hillier SL, Wiesenfeld HC, Heine RP (2000). Levels of vaginal secretory leukocyte protease inhibitor are decreased in women with lower reproductive tract infections. Am J Obstet Gynecol.

[B23] Piccioni PD, Kramps JA, Rudolphus A, Bulgheroni A, Luisetti M (1992). Proteinase/proteinase inhibitor imbalance in sputum sol phases from patients with chronic obstructive pulmonary disease. Suggestions for a key role played by antileukoprotease. Chest.

[B24] Abe T, Kobayashi N, Yoshimura K, Trapnell BC, Kim H, Hubbard RC, Brewer MT, Thompson RC, Crystal RG (1991). Expression of the secretory leukoprotease inhibitor gene in epithelial cells. J Clin Invest.

[B25] Saitoh H, Masuda T, Shimura S, Fushimi T, Shirato K (2001). Secretion and gene expression of secretory leukocyte protease inhibitor by human airway submucosal glands. Am J Physiol Lung Cell Mol Physiol.

[B26] Sallenave JM, Shulmann J, Crossley J, Jordana M, Gauldie J (1994). Regulation of secretory leukocyte proteinase inhibitor (SLPI) and elastase-specific inhibitor (ESI/elafin) in human airway epithelial cells by cytokines and neutrophilic enzymes. Am J Respir Cell Mol Biol.

[B27] van Wetering S, van der Linden AC, van Sterkenburg MA, Rabe KF, Schalkwijk J, Hiemstra PS (2000). Regulation of secretory leukocyte proteinase inhibitor (SLPI) production by human bronchial epithelial cells: increase of cell- associated SLPI by neutrophil elastase. J Investig Med.

[B28] Marchand V, Tournier JM, Polette M, Nawrocki B, Fuchey C, Pierrot D, Burlet H, Puchelle E (1997). The elastase-induced expression of secretory leukocyte protease inhibitor is decreased in remodelled airway epithelium. Eur J Pharmacol.

[B29] Abbinante-Nissen JM, Simpson LG, Leikauf GD (1993). Neutrophil elastase increases secretory leukocyte protease inhibitor transcript levels in airway epithelial cells. Am J Physiol.

[B30] Owen CA, Campbell MA, Boukedes SS, Campbell EJ (1997). Cytokines regulate membrane-bound leukocyte elastase on neutrophils: a novel mechanism for effector activity. Am J Physiol Lung Cell Mol Physiol.

[B31] Owen CA, Campbell MA, Sannes PL, Boukedes SS, Campbell EJ (1995). Cell surface-bound elastase and cathepsin G on human neutrophils: a novel, non-oxidative mechanism by which neutrophils focus and preserve catalytic activity of serine proteinases. J Cell Biol.

[B32] Navia MA, McKeever BM, Springer JP, Lin TY, Williams HR, Fluder EM, Dorn CP, Hoogsteen K (1989). Structure of human neutrophil elastase in complex with a peptide chloromethyl ketone inhibitor at 1.84-Å resolution. Proc Natl Acad Sci USA.

[B33] Grütter MG, Fendrich G, Huber R, Bode W (1988). The 2.5 A X-ray crystal structure of the acid-stable proteinase inhibitor from human mucous secretions analysed in its complex with bovine alpha-chymotrypsin. EMBO J.

[B34] Ma G, Greenwell-Wild T, Lei K, Jin W, Swisher J, Hardegen N, Wild CT, Wahl SM (2004). Secretory leukocyte protease inhibitor binds to annexin II, a cofactor for macrophage HIV-1 infection. J Exp Med.

[B35] Tseng CC, Tseng CP (2000). Identification of a novel secretory leukocyte protease inhibitor-binding protein involved in membrane phospholipid movement. FEBS Lett.

[B36] Perlmutter DH, Glover GI, Rivetna M, Schasteen CS, Fallon RJ (1990). Identification of a serpin-enzyme complex receptor on human hepatoma cells and human monocytes. Proc Natl Acad Sci USA.

[B37] Sallenave JM, M. ST, Cox G, Chignard M, Gauldie J (1997). Secretory leukocyte proteinase inhibitor is a major leukocyte elastase inhibitor in human neutrophils. J Leukoc Biol.

[B38] Schulze H, Korpal M, Bergmeier W, Italiano JE, Wahl SM, Shivdasani RA (2004). Interactions between the megakaryocyte/platelet-specific b1 tubulin and the secretory leukocyte protease inhibitor SLPI suggest a role for regulated proteolysis in platelet functions. Blood.

[B39] Martodam RR, Baugh RJ, Twumasi DY, Liener IE (1979). A rapid procedure for the large scale purification of elastase from human sputum. Preparative Biochemistry.

[B40] Veale CA, Bernstein PR, Bohnert CM, Brown FJ, Bryant C, Damewood JR, Earley R, Feeney SW, Edwards PD, Gomes B, Hulsizer JM, Kosmider BJ, Krell RD, Moore G, Salcedo TW, Shaw A, Silberstein DS, Steelman GB, Stein M, Strimpler A, Thomas RM, Vacek EP, Williams JC, Wolanin DJ, Woolson S (1997). Orally active trifluoromethyl ketone inhibitors of human leukocyte elastase. J Med Chem.

[B41] Dupuit F, Jacquot J, Spilmont C, Tournier JM, Hinnrasky J, Puchelle E (1993). Vectorial delivery of newly-synthesized secretory proteins by human tracheal gland cells in culture. Epithelial Cell Biol.

[B42] Macdonald SJ, Dowle MD, Harrison LA, Clarke GD, Inglis GG, Johnson MR, Shah P, Smith RA, Amour A, Fleetwood G, Humphreys DC, Molloy CR, Dixon M, Godward RE, Wonacott AJ, Singh OM, Hodgson ST, Hardy GW (2002). Discovery of further pyrrolidine trans-lactams as inhibitors of human neutrophil elastase (HNE) with potential as development candidates and the crystal structure of HNE complexed with an inhibitor (GW475151). J Med Chem.

[B43] de Garavilla L, Greco MN, Sukumar N, Chen ZW, Pineda AO, Mathews FS, Di Cera E, Giardino EC, Wells GI, Haertlein BJ, Kauffman JA, Corcoran TW, Derian CK, Eckardt AJ, Damiano BP, Andrade-Gordon P, Maryanoff BE (2005). A novel, potent dual inhibitor of the leukocyte proteases cathepsin G and chymase: molecular mechanisms and anti-inflammatory activity in vivo. J Biol Chem.

[B44] Park EY, Kim JA, Kim HW, Kim YS, Song HK (2004). Crystal structure of the Bowman-Birk inhibitor from barley seeds in ternary complex with porcine trypsin. J Mol Biol.

[B45] Levell J, Astles P, Eastwood P, Cairns J, Houille O, Aldous S, Merriman G, Whiteley B, Pribish J, Czekaj M, Liang G, Maignan S, Guilloteau JP, Dupuy A, Davidson J, Harrison T, Morley A, Watson S, Fenton G, McCarthy C, Romano J, Mathew R, Engers D, Gardyan M, Sides K, Kwong J, Tsay J, Rebello S, Shen L, Wang J, Luo Y, Giardino O, Lim HK, Smith K, Pauls H (2005). Structure based design of 4-(3-aminomethylphenyl)piperidinyl-1-amides: novel, potent, selective, and orally bioavailable inhibitors of betaII tryptase. Bioorg Med Chem.

[B46] Berman HM, Westbrook J, Feng Z, Gilliland G, Bhat TN, Weissig H, Shindyalov IN, Bourne PE (2000). The Protein Data Bank. Nucleic Acids Research.

[B47] Guex N, Peitsch MC (1997). SWISS-MODEL and the Swiss-PdbViewer: An environment for comparative protein modeling. Electrophoresis.

[B48] Llewellyn-Jones CG, Lomas DA, Stockley RA (1994). Potential role of recombinant secretory leucoprotease inhibitor in the prevention of neutrophil mediated matrix degradation. Thorax.

[B49] Kramps JA, Te Boekhorst AH, Fransen JA, Ginsel LA, Dijkman JH (1989). Antileukoprotease is associated with elastin fibers in the extracellular matrix of the human lung. An immunoelectron microscopic study. Am Rev Respir Dis.

[B50] Van Seuningen I, Aubert JP, Davril M (1992). Interaction between secretory leucoprotease inhibitor and bronchial mucins or glycopeptides. Physiopathological implications for the protection of mucins against proteolysis by human leucocyte elastase. Biochem J.

[B51] Miller KW, Evans RJ, Eisenberg SP, Thompson RC (1989). Secretory leukocyte protease inhibitor binding to mRNA and DNA as a possible cause of toxicity to Escherichia coli. J Bacteriol.

[B52] Bruch M, Bieth JG (1986). Influence of elastin on the inhibition of leucocyte elastase by a1-proteinase inhibitor and bronchial inhibitor. Biochem J.

